# Mother–offspring distances reflect sex differences in fine-scale genetic structure of eastern grey kangaroos

**DOI:** 10.1002/ece3.1498

**Published:** 2015-04-22

**Authors:** Wendy J King, Dany Garant, Marco Festa-Bianchet

**Affiliations:** 1 School of Biological Sciences, University of QueenslandSt. Lucia, Queensland, 4072, Australia; 2 Biology Department, Bishop's University2600 College St., Sherbrooke, Quebec, J1M 1Z7, Canada; 3 Département de biologie, Université de Sherbrooke2500 boul. de l'Université, Sherbrooke, Quebec, J1K 2R1, Canada; 4 School of BioSciences, The University of MelbourneMelbourne, Victoria, 3010, Australia

**Keywords:** Philopatry, relatedness, sex-biased dispersal, spatial autocorrelation

## Abstract

Natal dispersal affects life history and population biology and causes gene flow. In mammals, dispersal is usually male-biased so that females tend to be philopatric and surrounded by matrilineal kin, which may lead to preferential associations among female kin. Here we combine genetic analyses and behavioral observations to investigate spatial genetic structure and sex-biased dispersal patterns in a high-density population of mammals showing fission–fusion group dynamics. We studied eastern grey kangaroos (*Macropus giganteus*) over 2 years at Wilsons Promontory National Park, Australia, and found weak fine-scale genetic structure among adult females in both years but no structure among adult males. Immature male kangaroos moved away from their mothers at 18–25 months of age, while immature females remained near their mothers until older. A higher proportion of male (34%) than female (6%) subadults and young adults were observed to disperse, although median distances of detected dispersals were similar for both sexes. Adult females had overlapping ranges that were far wider than the maximum extent of spatial genetic structure found. Female kangaroos, although weakly philopatric, mostly encounter nonrelatives in fission–fusion groups at high density, and therefore kinship is unlikely to strongly affect sociality.

## Introduction

Natal dispersal is a fundamental parameter in life history and population biology and affects gene flow (Slatkin 1987; Garant et al. 2007). Sex-biased dispersal may be caused by inbreeding avoidance and/or benefits gained by one sex from increased access to resources or mates (Greenwood 1980; Pusey 1987), while factors promoting philopatry include benefits of familiarity with the natal area and kin cooperation (Lawson Handley and Perrin 2007). In mammals, dispersal is usually biased toward males, with females tending to be philopatric and thus surrounded by matrilineal kin (Greenwood 1980; Michener 1983; Lawson Handley and Perrin 2007) that may behave cooperatively (Hamilton 1964).

Fine-scale genetic structuring has been found among females in several species of mammals, for example black rhinoceros *Diceros bicornis* (Van Coeverden de Groot et al. 2011), white-tailed deer *Odocoileus virginianus* (Cullingham et al. 2011), red deer *Cervus elaphus* (Pérez-Espona et al. 2010), black bears *Ursus americanus* (Roy et al. 2012), bobcats *Lynx rufus* (Croteau et al. 2010), and Richardson's ground squirrels *Spermophilus richardsonii* (van Staaden et al. 1996). In contrast, genetic structuring is absent in other species, for example American badgers *Taxidea taxus* (Kierepka et al. 2012), Siberian lemmings *Lemmus sibericus* (Ehrich and Stenseth 2001), degu *Octogon degus* (Quirici et al. 2011) and inconsistent in rabbits *Oryctolagus cuniculus* (Richardson et al. 2002). Although allele frequencies provide information on average gene flow over many generations in the recent past, observed dispersal tendencies and distances provide complementary information on the extent and direction of movements at a particular time when environmental conditions may also be assessed (Slatkin 1987; Lawson Handley and Perrin 2007). Sex-biased dispersal appears to be an important catalyst of social evolution from multimale multifemale aggregates to stable cooperative groups (Schultz et al. 2011). The extent of female philopatry in species showing flexible spatiotemporal grouping patterns (‘fission–fusion’ dynamics (Aureli et al. 2008)) is thus of interest.

Previous fine-scale studies of dispersal in large mammals in fission–fusion societies have focused on ungulates such as African savannah elephants *Loxodonta africana* (Archie et al. 2008), forest elephants *Loxodonta cyclotis* (Schuttler et al. 2014), wild boar *Sus scrofa* (Poteaux et al. 2009), red deer (Nussey et al. 2005), white-tailed deer (Mathews and Porter 1993), and domestic sheep *Ovis aries* (Coltman et al. 2003; Nituch et al. 2008). In all these species, females and males follow the typical mammalian male-biased dispersal pattern with persistent kin associations among adult females.

Little is known about dispersal in marsupials. Asocial common wombats *Vombatus ursinus* and semi-social southern hairy-nosed wombats *Lasiorhinus latifrons* are unusual among mammals in showing female-biased dispersal, which may occur postbreeding (Banks et al. 2002; Walker et al. 2008). Both sexes can be philopatric in communally nesting agile antechinus *Antechinus agilis* (Banks et al. 2005). Females, but not males, are philopatric in semi-social mountain brushtail possums *Trichosurus cunninghami* (Blyton et al. 2014). Brush-tailed rock wallaby *Petrogale penicillata* females show strong philopatry (Hazlitt et al. 2004) in a social system with discrete multimale multifemale groups (Laws and Goldizen 2003). To date, however, there have been no detailed studies of dispersal in marsupials that live in fission–fusion groups.

Eastern grey kangaroos *Macropus giganteus* are large marsupials that can form extensive aggregations in open habitat (Jaremovic and Croft 1991). The social structure is fission–fusion, with individuals frequently joining and leaving groups that typically range in size between 3 and 10 individuals (Jarman and Coulson 1989). Only weak genetic structuring has been found at scales over 20 km, although range-wide genetic analyses showed that more dispersers are male than female (Zenger et al. 2003). Females are generally thought to be sedentary but long-range movements (>12 km) postbreeding have been documented (Jarman and Taylor 1983; Coulson et al. 2014). A recent study suggested that females were highly philopatric at a fine scale and exhibited preferential behavior toward kin (Best et al. 2013, 2014). That study, however, did not examine patterns in males, pooled 2 years of observations, and did not distinguish adults from subadults; therefore, subadult females were likely sampled while they were still closely associated with their mother. It is thus unclear to what extent adult females are philopatric and show positive genetic structuring at a fine scale in this species showing fission–fusion dynamics. Our aim was to determine the role of dispersal and settlement patterns in the genetic structure of both males and females in a population of eastern grey kangaroos at high density.

We first described the fine-scale genetic structure of eastern grey kangaroos for adults of both sexes using spatial autocorrelation analyses. To contrast observational data to genetic structure, we examined distances between mothers and offspring as the latter aged from permanent pouch emergence to sexual maturity and beyond. In addition, we compared dispersal tendencies of young males to those of young females. Finally, we interpreted results from spatial autocorrelation analyses in light of individual range sizes.

## Materials and Methods

We studied eastern grey kangaroos at Wilsons Promontory National Park, Australia (38^°^57′S, 146^°^17′E), from April 2010 to June 2012, as part of a long-term monitoring program that started in 2008. Kangaroos inhabit a 110-ha study area that consists of meadows surrounding a grassy landing strip. The area is mostly open with occasional trees and bushes such as coast tea-tree *Leptospermum laevigatum*, coast wattle *Acacia longifolia,* and coast banksia *Banksia integrifolia* (Davis et al. 2008). Densities of kangaroos were high (approximately 6 individuals/ha) both years (Glass 2013). We captured and marked about 50% of adult females and 80% of adult males following King et al. (2011). Animals were aged at first capture according to their mass and reproductive status (presence of pouch young or extended teats in females). Adult females weighed 20–35 kg and adult males 36–63.5 kg. We determined mother–offspring relationships by capturing young in the pouch or as suckling young-at-foot. Known adult mother–daughter pairs had been monitored from when the daughters were caught as pouch young and genetic relationships were confirmed using pairwise relatedness coefficients (see below). We estimated birthdates of pouch young based on hind leg, hind foot, and head lengths according to Poole et al. (1982). This species reaches sexual maturity in captivity at 2 years in females and 4 years in males (Poole and Catling 1974). Only one of 30 females first caught as pouch young reproduced before 3 years of age; that female was classed as an adult, but other 2-year-old females were not. Subadults were mostly 22–30 months old (2-year-olds) in October through June and young-at-foot were 10–18 months old (1-year-olds). For some analyses of observational data, we separated males into a small male category (25–36 kg; 3- and 4-year-olds) and large adult males (>38 kg; at least 5 years old).

A small tissue sample (approximately 2 mm diameter) was collected from the ear of each individual at first capture, preserved in 95% ethanol, and refrigerated at 4°C until laboratory analyses. DNA extractions were carried out using the salting-out protocol described in Chambers and Garant (2010). DNA concentration was initially determined for each sample by gel electrophoresis and diluted to a final concentration of 5 ng/*μ*L for polymerase chain reaction (PCR) amplification.

Microsatellite amplification was performed at nine loci using a GeneAmp PCR System 9700 thermocycler (Applied Biosystems, Foster City, CA). Multiplex PCR conditions and reaction mixture recipes are provided in Tables S1 and S2. PCR products were visualized using an AB 3130× capillary DNA sequencer (Applied Biosystems) by adding 0.15 *μ*L GeneScan 600 LIZ (Applied Biosystems) internal size standard and 8.35 *μ*L Hi-Di Formamide (Applied Biosystems) to 1.5 *μ*L total PCR products. Allele size was assessed using GeneMapper version 4.1 (Applied Biosystems).

We used KINGROUP v2 (Konovalov et al. 2004) to calculate observed and expected heterozygosity and deviation from Hardy–Weinberg equilibrium at each locus. Pair-wise relatedness coefficients (*r*) were estimated in KINGROUP as per KINSHIP (Queller and Goodnight 1989; Goodnight and Queller 1999). To check for independence of loci, we tested for linkage disequilibrium using log likelihood ratios in GENEPOP 4.2 (Raymond and Rousset 1995; Rousset 2008).

Observations of marked individuals took place using 8 × 32 binoculars (Leica, Wetzlar, Germany) for 9–15 days per month from April 2010 to March 2012, occurring while most individuals were feeding for about 2.5 hours after dawn and before dusk. Additional observations occurred on 5 days per month from May to June 2012. Individuals’ locations were recorded on foot from a distance of approximately 15 m using a hand-held Global Positioning System unit (GPSmap 60Cx, Garmin, Olathe, KS) with a precision of 4 m, and adjusted for observer/animal distance using a range-finder (SCOUT1000, Bushnell, Lenexa, KS) and compass (KB-14/360R, Suunto, Keili, Finland). Only the first location of an individual each day was used, to avoid temporal autocorrelation (Swihart and Slade 1985, 1997). Observations of adults were divided into 2 years: 15 April 2010 to 16 March 2011 and 5 April 2011 to 10 March 2012.

We used locations of individuals during foraging periods to calculate foraging range and core area sizes as 95% and 50% fixed kernels (Worton 1989). We employed Ranges8 version 2.5 (Kenward et al. 2008) and a smoothing factor *h* of 0.63, which was obtained as the median using least-squares cross-validation (Kenward 2001), and a minimum of 30 locations per individual (Seaman et al. 1999). The size of cumulative 95% kernels approached an asymptote at 25 locations. Very occasionally, individuals made excursions from their foraging range (defined as being seen at a location outside the 110-ha area that was at least 500 m from any other location for that individual, usually along a road or track) and these sightings were excluded from analyses (12 of 6876 sightings involving six adults in 2010–2011 and 15 of 8043 sightings involving six adults in 2011–2012), including the range size calculations. A few sightings by Park staff were verified to establish individual identities. Because we only observed kangaroos while they were foraging, we possibly underestimated their home range sizes; however, kangaroos usually rested in the same areas as where they foraged and our observations included the 3-h period after dawn, when kangaroo movements are greatest (Clarke et al. 1989). More adult females (72% of 106 individuals) than males (50% of 50 individuals) observed in the first year were also observed in the second year. Adult females were seen on average 62.4 ± 2.6 times in 2010–2011 and 57.8 ± 2.4 times in 2011–2012; adult males were seen 44.8 ± 4.0 times in 2010–2011 and 38.0 ± 2.9 times in 2011–2012. Many adult males were seen only 10–29 times per year (15 of 39 individuals in 2010–2011 and 19 of 50 individuals in 2011–2012) and so we did not use kernel analysis to estimate centers of activity, in order to maximize the number of individuals.

We calculated centroids as mean *x*, *y* coordinates for adults seen at least 10 times on the study area in either year. Next, we calculated centroids for adults seen both years as the overall mean *x*, *y* coordinates if seen at least 10 times in either year. Similarly, we calculated centroids for mothers and their offspring seen at least 10 times during 4-month periods when offspring were 10 to 41 months of age. Individuals were seen 20.6 ± 0.3 times per 4-month period.

We used the program GenAlEx 6.5 (Peakall and Smouse 2006, 2012) to calculate matrices of pairwise genetic distances among adults according to Peakall et al. (1995) and Smouse and Peakall (1999). Genetic distance matrices for each locus were summed across all loci under the assumption of independence to generate a total genetic distance matrix. We then constructed a geographic distance matrix consisting of the Euclidean distance (in meters) between all pairs of centroids. To analyze global autocorrelation, the total genetic distance matrix was compared to the geographic distance matrix using a Mantel (1967) test and 9999 random permutations. Analyses were first conducted on the overall dataset and then for each sex and year separately.

To investigate local autocorrelation, we correlated genetic distances with geographic distances at increasing distance classes of 15, 20, 25, 50, 75, and 100 m in GenAlEx 6.5 using 9999 permutations to determine confidence intervals and 10,000 bootstrap resampling for standard errors and plotted the resulting correlograms. We chose these distance classes based on pairwise sample sizes and a previous study that found positive genetic structure up to 80 m among females (Best et al. 2014). Previous studies have often used relatedness coefficients rather than genetic distance to describe genetic structure of mammalian populations (Lawson Handley and Perrin 2007), thus we also briefly describe the relationship between relatedness and geographic distance when a positive genetic structure was detected in the initial analysis.

To assess factors affecting distances between centroids of mothers and known offspring, we used linear mixed-effects models in the R environment version 2.15.2 (R Development Core Team 2012) with offspring identity included as a random factor. Mother identity was also initially included as a random factor in the model but was not significant. Offspring age (linear and measured in 4-month periods), offspring sex (factor), and year (factor) were included as fixed effects in models. Distance between centroids was first transformed using log(*x* + 1) to assure normal distribution of model residuals. We sequentially removed the least significant parameter (based on its *P*-value, threshold ≥0.05) from the model using stepwise backward selection (Crawley 2007). Differences between logarithmically transformed range sizes of different age–sex classes were assessed using ANOVA and Bonferroni post hoc tests.

We used *t*-tests to compare the distances over which adult males and females moved their centroids from 1 year to the next. Distances moved were transformed (log) to ensure normal distribution and results are presented back-transformed. Proportions of males and females that dispersed (born on the study area and seen at least 750 m from their original place of capture) were compared using Fisher Exact tests and calculated for three cohorts (birthdates September–May 2006/2007, 2007/2008, and 2008/2009) monitored from August 2008 to August 2014. Dispersing individuals were opportunistically sighted along roads and tracks. We chose 750 m as the minimum distance for dispersal because, as noted above, individuals were rarely seen more than 500 m from another of their own locations and no subadults or small males were seen at distances between 574 and 810 m from their original place of capture. Because we did not always have sufficient observations to calculate centroids of dispersing individuals, we used the original place of capture, which was as a pouch young on 70% of occasions, to measure dispersal distances. We employed Mann–Whitney *U*-tests to compare distances between centroids of mothers and known sons or daughters at different ages and to compare dispersal distances of males vs. females. Because the distance between known offspring and their mothers did not appear to increase linearly as they aged, we fitted quadratic lines to the data and compared the fit to linear models using extra sum-of-squares *F*-tests. We then chose the nonlinear model if it differed significantly (*P *<* *0.05) from the linear model. We used Pearson correlations to compare distances between centroids of adults and their pairwise relatedness where genetic structure had been found and sample sizes were most robust, that is, for adult females within 25 m.

## Results

### Microsatellite analyses

The nine microsatellite loci were polymorphic and did not deviate from Hardy–Weinberg equilibrium (Table S3). Linkage disequilibrium occurred in 1 of 36 possible combinations of loci (2.8% of combinations; loci G26-4 and T3-1T; *P *<* *0.001), and therefore spatial genetic autocorrelation analyses were repeated excluding G26-4. Results were quantitatively similar, and thus we present analyses using the 84 alleles across all nine loci (Table S3).

### Spatial autocorrelation

**Figure 1:**
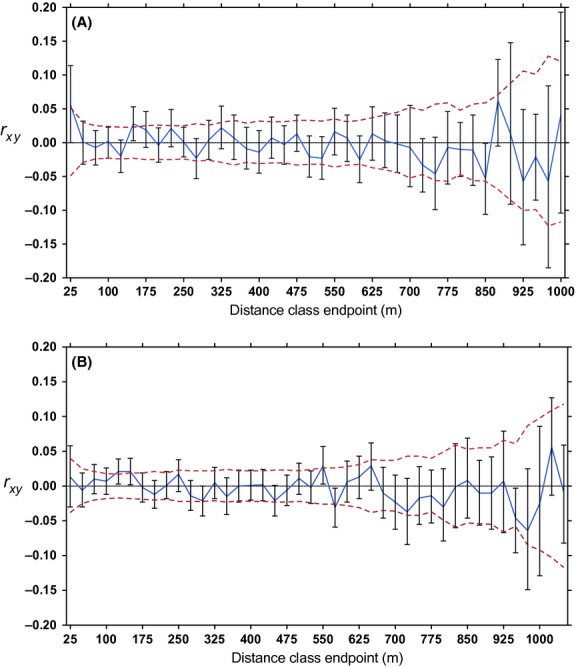
Correlograms for adult female eastern grey kangaroos at Wilsons Promontory National Park, Australia, in (A) 2010–2011 and (B) 2011–2012, comparing genetic distance to spatial distance in m, using 25-m distance classes. “*r*_*xy*_” refers to the correlation coefficient and is graphed in blue with 95% error bars from 10,000 bootstraps, while the 95% confidence intervals around *r*_*xy*_ - 0 are red-dashed lines.

Global spatial autocorrelation analyses revealed a weak positive spatial structure for females in both years (2010–2011, *r*_*xy*_ - 0.077, *P *-* *0.036 and 2011–2012, *r*_*xy*_ - 0.095, *P *-* *0.008; Table1). In contrast, males showed no genetic structure in either year (2010–2011, *r*_*xy*_ - 0.028, *P *-* *0.31 and 2011–2012, *r*_*xy*_ - 0.035, *P *-* *0.25; Table1). Maximum distances were 971 and 1363 m between male centroids and 1204 and 1337 m between female centroids for the 2 years, respectively. Using distance classes of 15, 20, and 25 m, local autocorrelation analyses showed significant spatial structure for females in the first year at the 0–15, 0–20, and 0–25 m classes and random beyond about 50 m (Table2). There were also weak positive correlations at 125–150 m (*r*_*xy*_ - 0.028, *P *-* *0.008, *n *-* *177), 200–225 m (*r*_*xy*_ - 0.021, *P *-* *0.047, *n *-* *154), and 850–875 m (*r*_*xy*_ - 0.062, *P *-* *0.044, *n *-* *19; Fig.1). Correlation coefficients for the first distance classes did not differ from zero for females the second year (Table2); however, there were weak positive correlations at 100–125 m (*r*_*xy*_ - 0.021, *P *-* *0.006, *n *-* *333), 125–150 m (*r*_*xy*_ - 0.021, *P *-* *0.015, *n *-* *269), and 525–550 m (*r*_*xy*_ - 0.029, *P *-* *0.012, *n *-* *144; Fig.1). The spatial structure for males was not significant in any of the distance classes starting at zero either year (Table3).

**Table 1:** Results of global autocorrelations between genetic and geographic distances for adult female and male eastern grey kangaroos at Wilsons Promontory National Park, Australia, 2010–2011, 2011–2012, and both years combined; *r*_*xy*_ - correlation coefficient according to Mantel tests; *P *- probability with 9999 permutations; *n *- sample size. Significant correlations are in bold

Sex	Year	*r*_*xy*_	*P*	*n*
Female	2010–2011	**0.077**	**0.036**	82
	2011–2012	**0.095**	**0.008**	106
	Both	**0.079**	**0.012**	112
Male	2010–2011	0.028	0.31	39
	2011–2012	0.035	0.25	50
	Both	0.024	0.30	64
All adults	2010–2011	0.030	0.19	121
	2011–2012	**0.059**	**0.030**	156
	Both	0.030	0.14	176

**Table 2:** Results of autocorrelations between genetic and geographic distances for adult female eastern grey kangaroos in increasing distance classes at Wilsons Promontory National Park, Australia, 2010–2011 and 2011–2012; *r*_*xy*_ - correlation coefficient; *P *- probability with 9999 permutations; *n *- number of pairs in each distance class. The *x*-intercept was set to zero if the correlation was not significant (*P *>* *0.05). Significant correlations are in bold

	2010–2012				2011–2012			
Distance class	*r*_*xy*_	*P*	*n*	Intercept (m)	*r*_*xy*_	*P*	*n*	Intercept (m)
0–15 m	**0.077**	**0.023**	18	51.0	0.017	0.30	23	0
0–20 m	**0.073**	**0.010**	29	47.1	0.015	0.26	46	0
0–25 m	**0.056**	**0.019**	38	53.2	0.013	0.27	66	0
0–50 m	0.016	0.12	137	0	−0.001	0.51	217	0
0–75 m	0.004	0.30	289	0	0.005	0.25	415	0
0–100 m	0.003	0.29	449	0	0.006	0.14	738	0

**Table 3:** Results of autocorrelations between genetic and geographic distances for adult male eastern grey kangaroos in increasing distance classes at Wilsons Promontory National Park, Australia, 2010–2011 and 2011–2012; *r*_*xy*_ - correlation coefficient; *P *- probability with 9999 permutations; *n *- number of pairs in each distance class. The *x*-intercept was set to zero if the correlation was not significant (*P *>* *0.05)

	2010–2012				2011–2012			
Distance class	*r*_*xy*_	*P*	*n*	Intercept (m)	*r*_*xy*_	*P*	*n*	Intercept (m)
0–15 m	−0.040	0.67	4	0	0.082	0.18	3	0
0–20 m	−0.046	0.73	5	0	0.082	0.18	3	0
0–25 m	−0.039	0.71	6	0	0.051	0.26	4	0
0–50 m	−0.007	0.58	23	0	0.010	0.40	13	0
0–75 m	0.000	0.50	38	0	−0.002	0.52	33	0
0–100 m	0.009	0.32	54	0	0.009	0.31	66	0

### Observational data

**Figure 2:**
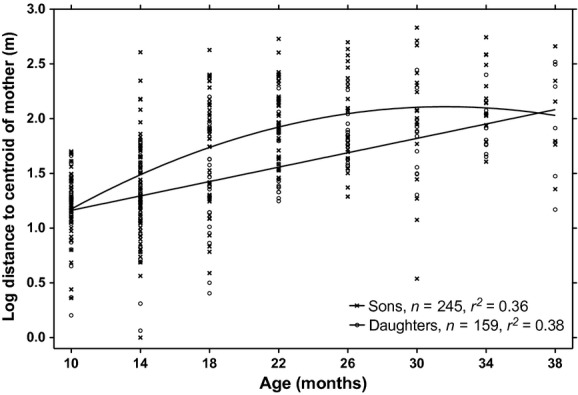
Distance between centroids of 85 eastern grey kangaroo mothers and their 134 offspring for 4-month age periods starting at 10 months of age at Wilsons Promontory National Park, Australia, April 2010–June 2012. The solid line is a quadratic regression for sons (compared to a linear fit, *F*_1,242_ - 19.46, *P *<* *0.001); the dotted line is a linear regression for daughters.

**Figure 3:**
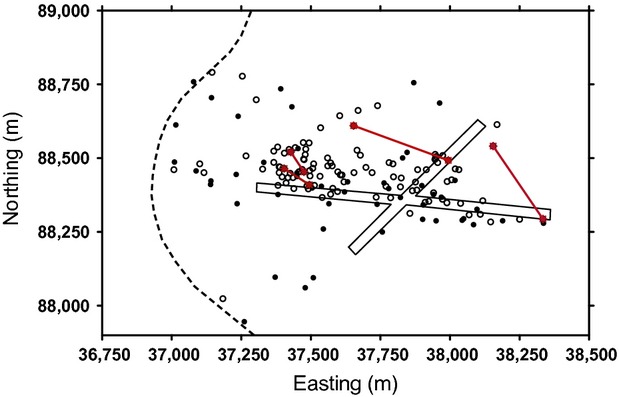
Map of 50 adult male (closed circle) and 106 adult female (open circle) centroids of marked eastern grey kangaroos at Wilsons Promontory National Park, Australia, 2011–2012. The red lines connect centroids of 4 known mother–daughter pairs, the dashed line indicates the main road, and the solid lines outline the arms of the grassy airstrip.

Adult males moved their centroid farther from 1 year to the next than did adult females (178 ± 31 m vs. 79 ± 9 m, *t*_24,75_ - 4.13, *P *<* *0.001), indicating that they were less sedentary than females. Mothers shifted their centroid by 75 ± 5 m on average from one 4-month period to the next (*n *-* *81). Although offspring of both sexes were near their mothers at young ages, sons were located much farther from their mothers than were daughters between the ages of 18 and 25 months (Table4, Fig.2). Daughters also moved away from their mothers as they aged but appeared to delay this movement until 26 months of age (Table4). In addition to the sex and age effects, distances between mother–offspring pairs were shorter the second year (Table5). Identity of the offspring contributed 23% to the overall variance in distances. Sizes of ranges varied according to sex-age class (95% kernel areas: ANOVA, *F*_6,204_ - 21.20, *P *<* *0.001; 50% kernel areas: ANOVA, *F*_6,204_ - 12.19, *P *<* *0.001; 95% kernel widths: ANOVA, *F*_6,204_ - 21.51, *P *<* *0.001; 50% kernel widths: ANOVA, *F*_6,204_ - 9.31, *P *<* *0.001; Table6). Adult females, subadult females, and young-at-foot of both sexes had 95% ranges with median width of 509–570 m. Large adult males and small males had much wider 95% ranges, with medians of 842 and 927 m, respectively. Subadult males had 95% ranges that were intermediate in width (median - 706 m). Median distance between the four known adult mother–daughter pairs was 206 m in 2011–2012 (Fig.3) so that three of four pairs overlapped their 95% ranges and two of four pairs overlapped their 50% core areas. Pairwise relatedness (*r*) between adult mother–daughter pairs ranged from 0.407 to 0.648. There were no known adult mother–daughter pairs in the first year.

**Table 4:** Median distance (m) between the centroids of 85 eastern grey kangaroo mothers and their offspring of different ages at Wilsons Promontory National Park, Australia, April 2010–June 2012

Age	Sons (*n*)	Daughters (*n*)	Mann–Whitney *U*	*P*
10–13 months	17 (47)	17 (34)	737.5	0.56
14–17 months	19 (53)	20 (36)	822.5	0.27
18–21 months	77 (45)	23 (23)	329.0	0.015
22–25 months	108 (38)	27 (19)	99.5	0.001
26–29 months	93 (24)	62 (16)	152.5	0.28
30–33 months	117 (17)	75 (13)	76.5	0.16
34–37 months	138 (15)	81 (11)	48.0	0.08
38–41 months	102 (6)	81 (7)	20.0	0.94

**Table 5:** Final model of a general linear mixed model of distances between centroids (log-transformed) of 85 mothers and 134 offspring for eastern grey kangaroos at Wilsons Promontory National Park, Australia, April 2010–June 2012, *n *-* *404

Coefficient	Estimate	Standard error	*t*	*P*
Intercept	1.294	0.058	22.35	<0.001
Sex (female)	−0.038	0.088	−0.43	0.66
Year (2011–2012)	−0.142	0.042	−3.42	0.001
Age (14–17 months)	0.182	0.072	2.52	0.012
Age (18–21 months)	0.555	0.077	7.18	<0.001
Age (22–25 months)	0.802	0.082	9.80	<0.001
Age (26–29 months)	0.836	0.098	8.51	<0.001
Age (30–33 months)	0.845	0.111	7.59	<0.001
Age (34-37 months)	0.991	0.116	8.56	<0.001
Age (38–41 months)	0.866	0.165	5.24	<0.001
Sex (female) × Age (14–17 months)	−0.117	0.112	−1.04	0.30
Sex (female) × Age (18–21 months)	−0.294	0.126	−2.34	0.020
Sex (female) × Age (22–25 months)	−0.370	0.134	−2.76	0.006
Sex (female) × Age (26–29 months)	−0.157	0.148	−1.07	0.29
Sex (female) × Age (30–33 months)	−0.169	0.164	−1.03	0.30
Sex (female) × Age (34–37 months)	−0.230	0.173	−1.33	0.18
Sex (female) × Age (38–41 months)	−0.035	0.227	−0.16	0.88

**Table 6:** Mean range sizes and widths based on 95% and 50% kernels for eastern grey kangaroos of different sex-age classes at Wilsons Promontory National Park, Australia, October 2010–June 2011. Sex-age classes with the same superscript did not differ in range size according to ANOVA and post hoc Bonferroni multiple comparison tests on log-transformed data (*P *>* *0.05)

		95% kernel				50% kernel			
Sex-age class	*n*	Size (ha)	SE	Width (m)	SE	Size (ha)	SE	Width (m)	SE
Small male	9	30.9^a^	4.2	933^a^	62	10.1^a^	1.7	646^a^	85
Large adult male	30	27.3^a^	1.9	859^ab^	35	8.7^a^	0.5	515^ab^	23
Subadult male	25	20.6^ab^	1.5	714^bc^	28	7.1^ab^	0.7	426^bc^	27
Subadult female	18	15.4^bc^	1.4	624 ^cd^	37	5.0^bc^	0.6	367^c^	31
Adult female	82	13.6^c^	0.6	585^d^	15	4.8^c^	0.3	375^c^	14
Young-at-foot female	19	12.3^c^	1.1	560^d^	25	4.2^c^	0.5	350^c^	28
Young-at-foot male	28	11.3^c^	0.8	526^d^	20	4.1^c^	0.3	333^c^	17

Mean relatedness (*r*) among adult females was low both years (−0.013 ± 0.002 in 2010–2011 (*n *-* *6642 pairs) and −0.010 ± 0.002 in 2011–2012 (*n *-* *11,130 pairs)) and only weakly positive for those pairs of females with centroids within 25 m (0.065 ± 0.028, *n *-* *38 in 2010–2011 and 0.028 ± 0.022, *n *-* *66 in 2011–2012, Fig.4), confirming results of the local spatial autocorrelation analyses. Median distance between centroids of pairs of highly related adult females (relatedness coefficient *r *>* *0.45) was 268 m (*n *-* *25) in 2010–2011 and 243 m (*n *-* *42) in 2011–2012. The distribution of pairwise distances of adult females was such that 33% occurred in the 0–200 m classes, 9% in the 200–250 m class, and 58% in the >250 m classes. Mean relatedness (*r*) among adult males was −0.026 ± 0.005 in 2010–2011 (*n *-* *1406 pairs) and −0.020 ± 0.003 in 2011–2012 (*n *-* *2450 pairs).

**Figure 4:**
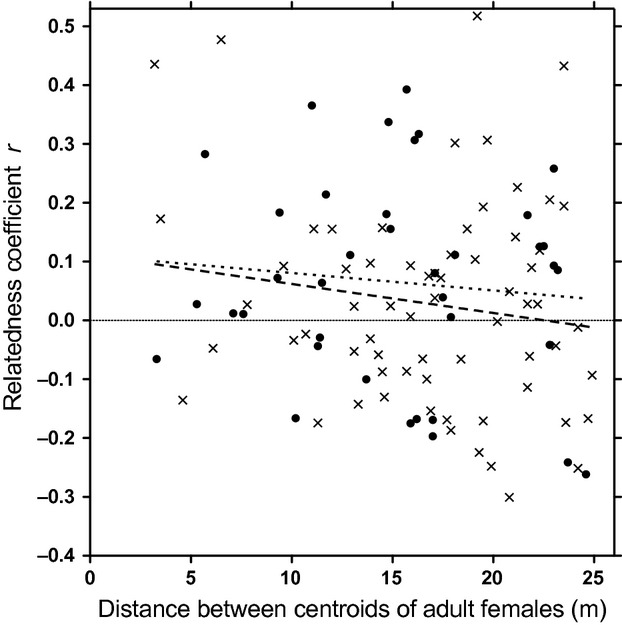
Pairwise relatedness coefficients *r* plotted against distance between centroids for adult female eastern grey kangaroos that had centroids within 25 m at Wilsons Promontory National Park, Australia, 2010–2011 (closed circles, *n *-* *38) and 2011–2012 (crosses, *n *-* *66). The dotted and dashed lines represent Pearson correlations for the 2 years (*r*_*p*_ - 0.10, *P *-* *0.56 and *r*_*p*_ - 0.15, *P *-* *0.23, respectively).

In total, 20 subadults and young adults (18 males and 2 females) were known to disperse from the study area. More males than females dispersed (34% of 53 males vs. 6% of 34 females, Fisher Exact Test, *P *-* *0.003), but there was no difference in the median distance moved (2019 vs. 2486 m, respectively, *U *-* *15.0, *P *-* *0.84, *n* - 17 vs. 2). The greatest recorded dispersal distance was 4009 m. For 13 males of known birthdate, dispersal occurred at 24–63 months of age (mean - 43.3 ± 2.8 months). The single known-aged female that dispersed was 31 months old.

## Discussion

We found weak but significant fine-scale genetic structure among adult female kangaroos in both years, but none among adult males either year. Immature male kangaroos moved away from their mothers at a younger age than did immature females. Also, a higher proportion of males than females were observed to disperse.

Nonrandom spatial grouping of female kin can result from sex-biased dispersal and social segregation without active preference for kin associates (Coltman et al. 2003). A previous study of eastern grey kangaroos found that females were philopatric at the fine scale, but that female associations were only weakly related to kinship (Best et al. 2014). Because subadult females were combined with adult females in that study, however, it is unclear to what extent the associations were influenced by immature daughters, which often associate closely with their mothers. We restricted our analyses to adult females and found weak spatial genetic structure, even though females were sedentary from 1 year to the next. Because we combined behavioral observations with genetic analyses, we have an improved understanding of how the genetic structure among adult females arose. Adult daughters settled about 200–250 m away from their mothers but as one third of adult female pairs had centroids that were less than 200 m apart, the high density and adult survival rates affected temporal and spatial overlap of close kin such that females were unlikely to have close relatives as nearest neighbors. The positive genetic structure among females was thus likely a passive result of density and dispersal rather than active association.

Mammalian population genetic structure may be influenced by the extent of polygynous mating in addition to dispersal patterns (Stortz 1999). A rapid decline in fine-scale genetic structure was detected for red deer in conjunction with a sharp decrease in polygyny at high densities (Nussey et al. 2005). Male mating skew may be considerable in eastern grey kangaroos, with dominant males siring approximately 50% of juveniles in any 1 year in a population of about 55 individuals in a semi-captive environment (Miller et al. 2010). This high level of polygyny should result in daughters within a cohort being closely related through their fathers. The very restricted extent of genetic structuring found among the sedentary adult females in our study, however, indicates that male mating skew is likely much lower in free-ranging populations at high density.

Dispersal distances in mammals tend to correlate positively with body size but to be shorter for herbivorous species, so despite their relatively large size, kangaroos are expected to disperse a median distance of about 3–5 km (Sutherland et al. 2000). Range size is likely a better predictor of dispersal distance than body size, with the median distance dispersed predicted to be 4.0–5.9 km based on adult ranges of 570–842 m width (Bowman et al. 2002). Kangaroos in our study appeared to disperse about 2–2.5 km but because we only collected opportunistic sightings along roads and tracks we may have missed long-distance dispersal events.

Dispersal distances can be affected by environmental conditions that likely vary from year to year (Slatkin 1987). Environmental effects influenced dispersal distances in a solitary rodent *Tamias striatus* such that females dispersed farther in years of favorable resource conditions and thereby increased the extent of positive genetic structure (Dubuc-Messier et al. 2012). Critically, the maximum extent of spatial genetic structure for chipmunk females occurred at a scale (50–250 m) that approximated or exceeded the diameter of individual home ranges (40 m) so that neighbors were likely to be close relatives (Dubuc-Messier et al. 2012). In contrast, female eastern grey kangaroos live in fission–fusion societies with overlapping ranges that are far wider (around 550 m) than the maximum extent of spatial genetic structure (50 m). As a result, female kangaroos in our population must mostly encounter nonkin. Any preferential associations found after accounting for range overlap will thus likely depend on factors other than kinship, such as reproductive state (Jarman and Southwell 1986) or sociability (Réale et al. 2007).

## Data Accessibility

Spreadsheets of data have been archived at Dryad Digital Depository http://dx.doi.org/10.5061/dryad.b999q, including 1) genotypes and mean *x, y* coordinates for adult kangaroos by sex each year, 2) distances between mothers and young of different ages and sexes, 3) range sizes of 95% and 50% kernels for different sex-age classes, and 4) known dispersers with sex, age, and distance moved.

## Supporting Information

**Table S1.** Microsatellite loci details for multiplex PCR amplification. **Table S2.** Four polymerase chain reaction recipes for the nine microsatellite loci used in Table S1. **Table S3.** Size range, number of alleles, observed (*H*_o_) and expected (*H*_e_) heterozygosity, and probability of deviation from Hardy-Weinberg equilibrium (*P* H-W) for nine microsatellite loci in 176 adult eastern grey kangaroos at Wilsons Promontory National Park, Australia, 2010–2012.
