# Supplementary material for: Mother–offspring distances reflect sex differences in fine-scale genetic structure of eastern grey kangaroos
Source: Ecol Evol. 2015 Apr 22;5(10):2084–94. doi: 10.1002/ece3.1498 (PMC4449761; doi:10.1002/ece3.1498)
Supplement: Supplementary file 1 [file ece30005-2084-sd1.docx]

Supplementary Table S1. Microsatellite loci details for multiplex PCR amplification.

| Locus | Genbank accession number | Annealing temperature (ºC) | No. of cycles | Dye | PCR recipe | Amount in multiplex (μl) | Multiplex No. |
| --- | --- | --- | --- | --- | --- | --- | --- |
| G12-6 | AF322622 | 54 | 25 | VIC | D | 2.0 | 2 |
| G16-1 | AF322624 | 56 | 25 | PET | D | 2.0 | 2 |
| G16-2 | AF322625 | 54 | 30 | PET | A | 2.0 | 1 |
| G19-1 | AF322626 | 56 | 30 | NED | B | 2.0 | 1 |
| G26-4 | AF322628 | 56 | 30 | VIC | B | 1.0 | 2 |
| G31-1 | AF322629 | 56 | 25 | NED | B | 1.5 | 2 |
| G31-3 | AF322630 | 56 | 30 | FAM | A | 1.5 | 2 |
| T3-1T | AF322644 | 56 | 27 | FAM | A | 1.0 | 1 |
| T32-1 | AF322654 | 54 | 30 | VIC | C | 1.0 | 1 |

Supplementary Table S2. Four polymerase chain reaction recipes for the 9 microsatellite loci used in Table S1. Concentrations are listed for a total reaction volume of 10μl.

| Product | A | B | C | D |
| --- | --- | --- | --- | --- |
| dNTPs (mM) | 0.2 | 0.2 | 0.2 | 0.2 |
| MgCl_2_ (mM) | 2.5 | 2.5 | 2.5 | 2.5 |
| 10X reaction buffer (500mM KCl, 100mM tris-HCL) | 1X | 1X | 1X | 1X |
| BSA (mg/ml) | 0.4 | 0.4 | 0.4 | 0.4 |
| Fluorescently labeled forward primer (μM) | 0.3 | 0.1 | 0.5 | 0.5 |
| Unlabeled reverse primer (μM) | 0.3 | 0.1 | 0.5 | 0.5 |
| AmpliTaq Gold DNA polymerase (U)  (Applied Biosystems) | 0.35 | 0.5 | 0.25 | 0.5 |
| DNA (ng) | 10 | 10 | 10 | 10 |

Supplementary Table S3. Size range, number of alleles, observed (H_o_) and expected (H_e_) heterozygosity and probability of deviation from Hardy-Weinberg equilibrium (*P* H-W) for 9 microsatellite loci in 176 adult eastern grey kangaroos at Wilsons Promontory National Park, Australia, 2010–2012.

| Locus | Size range (bp) | No. of alleles | H_o_ | H_e_ | *P* H-W |
| --- | --- | --- | --- | --- | --- |
| G12-6 | 70–120 | 6 | 0.784 | 0.780 | 0.87 |
| G16-1 | 140–190 | 6 | 0.812 | 0.708 | 0.13 |
| G16-2 | 110–140 | 8 | 0.841 | 0.794 | 0.89 |
| G19-1 | 165–225 | 9 | 0.795 | 0.789 | 0.56 |
| G26-4 | 200–400 | 14 | 0.841 | 0.871 | 0.24 |
| G31-1 | 105–145 | 10 | 0.824 | 0.798 | 0.66 |
| G31-3 | 75–180 | 9 | 0.812 | 0.807 | 0.60 |
| T3-1T | 100–400 | 13 | 0.847 | 0.8829 | 0.44 |
| T32-1 | 140–195 | 9 | 0.744 | 0.713 | 0.78 |
| Mean |  | 9.3 | 0.811 | 0.788 |  |
